# Reliable Quantification of Powdered Ginger Adulteration by Vis–NIR Spectroscopy and Chemometrics

**DOI:** 10.3390/molecules31173091

**Published:** 2026-09-03

**Authors:** Rim Amine, Pablo F. Sánchez, Hala Kharkhour, Anas El-Laghdach, Miguel Palma, Latifa Azaroual

**Affiliations:** 1 Department of Chemistry, Faculty of Sciences, University Abdelmalek Essaadi, B.P. 2121, Mhannech II, Tetouan 93002, Morocco; rim.amine@etu.uae.ac.ma (R.A.); aellaghdach@uae.ac.ma (A.E.-L.); lazaroual@uae.ac.ma (L.A.); 2 Department of Analytical Chemistry, Faculty of Sciences, University of Cádiz, Agri-Food Campus of International Excellence (ceiA3), IVAGRO, 11510 Puerto Real, Spain; 3 Department of Chemical Metrology, National Institute of Industrial Technology (INTI), Buenos Aires B1650WAB, Argentina; pfsanchez@inti.gob.ar; 4 Stochastic and Deterministic Modeling Laboratory, Department of Mathematics, Faculty of Sciences Oujda, Mohammed First University, Oujda 60000, Morocco; hala.kharkhour.d24@ump.ac.ma

**Keywords:** food adulteration, Vis-NIR spectroscopy, ginger, chemometrics

## Abstract

Economically motivated adulteration of powdered ginger with low-cost cereal flours represents an increasing concern for food authenticity and quality control. The aim of this study was to develop and validate a rapid, reliable, and non-destructive method for the quantitative determination of powdered ginger adulteration using visible and near-infrared (Vis–NIR) spectroscopy coupled with chemometric modelling. Ginger powder samples were adulterated with wheat, corn, and rice flours at concentrations ranging from 5 to 50% (*w*/*w*), with particular emphasis on the low-to-medium adulteration interval (10–25%), where reliable quantification is especially relevant for food fraud detection. Spectral data acquired in the visible (400–700 nm), near-infrared (700–2500 nm), and combined Vis–NIR (400–2500 nm) regions were preprocessed using Savitzky–Golay filtering and evaluated using Partial Least Squares Regression (PLSR), Support Vector Regression (SVR), and Random Forest Regression (RFR). In addition, Linear Discriminant Analysis (LDA), Support Vector Machines (SVM), and Random Forest (RF) were compared for sample classification. Among the evaluated approaches, LDA achieved the highest classification accuracy (>95%) using the NIR spectroscopic region, while PLSR models developed from the NIR spectral region provided the best quantitative performance, with validation coefficients of determination above 0.99, prediction errors below 1%, and RPD values greater than 13. The results demonstrate that Vis–NIR spectroscopy combined with chemometric modelling enables accurate discrimination between authentic and adulterated samples, as well as reliable quantification of flour adulteration in powdered ginger without sample preparation or chemical reagents. The proposed methodology constitutes a rapid, environmentally friendly, and cost-effective analytical strategy with strong potential for routine quality control and food fraud prevention.

## 1. Introduction

Food adulteration encompasses deliberate practices intended to obtain an economic benefit, including the substitution or dilution of authentic ingredients, the addition of undeclared low-cost materials, false labelling, and misrepresentation of the geographical or botanical origin of a product. Food fraud represents a global concern because it undermines consumer confidence, causes substantial economic losses, distorts legitimate trade, and may pose serious risks to public health when undeclared allergens, toxic colorants, contaminants, or unsuitable raw materials are introduced into the food chain [1,2,3,4]. Although international regulatory frameworks establish requirements concerning food composition, traceability, labelling, and consumer protection, detecting fraudulent practices remains challenging, particularly in long and complex supply chains involving highly processed ingredients [5]. Spices are especially vulnerable because of their relatively high commercial value, extensive international trade, natural compositional variability, and frequent commercialization in ground or processed forms, in which the original morphological characteristics are no longer visible.

Several economically important spices, including saffron, paprika, turmeric, black pepper, vanilla, nutmeg, and ginger, have repeatedly been identified as products at risk of substitution, dilution, or mislabelling [5,6,7]. Ginger (Zingiber officinale Roscoe) is widely used as a culinary ingredient, flavouring agent, and traditional medicinal product. Its biological properties have been associated with antioxidant, anti-inflammatory, antiemetic, antimicrobial, and digestive effects [8,9]. The characteristic pungency and biological activity of ginger are mainly related to phenolic compounds, including gingerols, shogaols, paradols, and zingerone, while its aroma is associated with a complex volatile fraction containing mono- and sesquiterpenes [10]. Ginger also contains large proportions of carbohydrates, structural polysaccharides, proteins, lipids, and residual moisture. Once ginger is converted into powder, its visual authentication becomes difficult, and the product can be diluted with less expensive materials such as wheat, corn, rice, soybean, or chickpea flours; spent ginger material; other plant powders; starches; or artificial colorants. Such additions reduce commercial quality and may also introduce undeclared allergens, particularly when gluten-containing cereals or allergenic legumes are used [3,5].

Conventional methods for food-authenticity assessment include chromatographic, mass-spectrometric, molecular, immunological, microscopic, and stable-isotope techniques [11]. These methods may provide high sensitivity and chemical specificity, but they frequently require sample preparation, extraction procedures, trained personnel, laboratory infrastructure, relatively long analysis times, and the consumption of solvents and reagents. Vibrational spectroscopy, particularly near-infrared spectroscopy, has therefore attracted considerable attention as a rapid screening and quality-control technique. NIR spectroscopy is non-destructive, requires little or no sample preparation, generates virtually no chemical waste, and can simultaneously capture information associated with multiple constituents of a complex food matrix [12]. NIR absorption is mainly produced by overtones and combination vibrations involving C–H, O–H, and N–H bonds, which are abundant in carbohydrates, water, proteins, lipids, and phenolic compounds. However, the resulting bands are broad and strongly overlapping, making multivariate data analysis essential to extract analytically useful information from the spectra [12,13].

The visible region can provide complementary information associated with colour, pigments, particle characteristics, and light-scattering behaviour. Consequently, combining visible and NIR wavelengths may improve the detection of adulterants that differ from the authentic spice both chemically and physically [14]. Vis–NIR and NIR spectroscopy have already demonstrated considerable potential for food fraud detection in a wide variety of food products, particularly powdered matrices, owing to their ability to provide rapid, non-destructive chemical fingerprints suitable for authenticity assessment. Recent studies have successfully applied these techniques to detect and quantify adulteration in products such as paprika, cocoa powder, honey, and spices. For example, portable NIR spectroscopy combined with partial least squares discriminant analysis and regression successfully discriminated paprika powder adulterated with potato starch, acacia gum, and annatto, achieving prediction errors close to 2% for several adulterant mixtures [15]. Likewise, Vis–NIR spectroscopy coupled with chemometric modelling has recently been shown to accurately detect and quantify cocoa powder adulteration [16], while machine learning algorithms applied to Vis–NIR spectra have enabled the rapid and automated detection of honey adulteration [17]. In addition, NIR diffuse-reflectance spectra combined with random-forest one-class models successfully detected adulteration of ground nutmeg with chemically diverse materials, outperforming conventional PLS-DA and SIMCA models [18]. These studies demonstrate that spectroscopic fingerprints, particularly when combined with advanced chemometric and machine learning techniques, can reliably reveal food adulteration even when no single wavelength can be unequivocally assigned to a specific adulterant.

Chemometric modelling is therefore a central component of spectroscopic food authentication methods. Partial least squares regression is one of the most widely used algorithms because it efficiently handles highly collinear spectral variables and datasets in which the number of wavelengths greatly exceeds the number of samples. However, PLS is fundamentally a linear modelling technique, and the relationship between spectral responses and adulterant concentration may be affected by nonlinearities arising from particle size, scattering, moisture, ingredient interactions, and matrix heterogeneity [19]. In contrast, support vector methods are particularly useful for high-dimensional datasets and can model nonlinear responses through kernel functions, whereas random forests (RF) combine multiple decision trees and are comparatively resistant to noise, interactions, and overfitting when appropriately optimized. Nevertheless, no algorithm can be assumed to be universally superior; comparative modelling using independent validation samples is required to establish whether increased model complexity produces a genuine improvement over conventional PLS approaches [20].

The value of combining spectroscopy with advanced modelling has been demonstrated in several spice-authentication studies. Specifically for ginger, FT-NIR spectroscopy together with PCA, PLS-DA, and PLS regression has been used to identify ginger powder adulterated with soybean, corn, and wheat flours and to estimate the adulteration level [21]. More recently, NIR spectroscopy has been used to predict 6-gingerol content and discriminate ginger powders according to geographical origin, demonstrating that spectral analysis can provide information related not only to adulteration but also to relevant quality attributes and provenance [22].

Despite these advances, research specifically addressing the reliable quantification of powdered ginger adulteration remains limited, particularly when different cereal-flour adulterants, spectral intervals, preprocessing strategies, and linear and nonlinear regression methods are evaluated under the same experimental design. Therefore, the objective of this study was to develop and validate a rapid and non-destructive Vis–NIR spectroscopic method for quantifying powdered ginger adulteration with wheat, corn, and rice flours. Spectral information from the visible, NIR, and combined Vis–NIR regions was evaluated together with Savitzky–Golay preprocessing, the Boruta algorithm for variable selection, and three regression approaches—partial least squares regression, support vector regression, and random forest regression. Particular attention was paid to the predictive reliability of the models within the 10–25% adulteration interval, with the aim of establishing a simple and robust analytical strategy for routine ginger-authenticity assessment and food-fraud control.

## 2. Results and Discussion

Samples, including authentic powders and samples adulterated with wheat, corn, rice, or a mixture of the three flours, were analysed using Vis–NIR spectroscopy. The acquired spectra covered the wavelength range from 400 to 2500 nm, including both the visible (400–700 nm) and near-infrared (700–2500 nm) regions. The study aimed to evaluate the ability of these spectral regions, individually and in combination, to detect and accurately quantify different levels of adulteration.

An initial exploratory analysis was carried out to assess spectral quality, identify possible outliers, and evaluate the overall variability of the dataset before chemometric modelling. No anomalous samples requiring exclusion were detected, indicating good consistency in both sample preparation and spectral acquisition.

The chemical basis for the spectral discrimination between authentic and flour-adulterated ginger can be interpreted by considering the main absorption regions of plant-derived materials in the NIR range. NIR spectra are dominated by broad and overlapping absorption bands arising mainly from overtones and combinations of O–H, C–H, and N–H vibrations. Previous studies on ginger and related plant materials have identified relevant spectral features at approximately 1200, 1440–1570, 1740, 1930, and 2100–2320 nm [21]. The region around 1450 nm is mainly associated with the first overtone of O–H stretching, whereas the strong absorption around 1900–1950 nm is related to O–H combination vibrations and is particularly influenced by water and hydroxyl-containing constituents. Bands within approximately 1700–1800 nm are associated mainly with C–H first overtones, while the 2000–2350 nm region contains several combination bands involving O–H, C–H, N–H, and C–O vibrations associated with carbohydrates, proteins, and other organic constituents. These regions are therefore particularly relevant for differentiating plant-derived materials with different chemical compositions.

The incorporation of wheat, corn, or rice flour is expected to modify these spectral regions because cereal flours are rich in starch, composed primarily of amylose and amylopectin, whereas ginger represents a chemically more complex matrix containing carbohydrates together with proteins, lipids, phenolic compounds, essential-oil constituents, and structural polysaccharides. Consequently, replacing part of the ginger matrix with cereal flour progressively modifies the relative contributions of O–H-, C–H-, and C–O-related absorptions, particularly in the carbohydrate-sensitive NIR regions. Therefore, the adulteration is expected to produce systematic variations across several correlated wavelengths, generating a multivariate spectral fingerprint that can be exploited by chemometric models.

### 2.1. Classification of Ginger Samples Using Linear Discriminant Analysis (LDA), Support Vector Machines (SVMs), and Random Forest (RF)

The ability of the Vis–NIR spectral data to discriminate authentic ginger samples from those adulterated with different cereal flours was first evaluated using supervised classification models. Five sample categories were considered: authentic ginger, ginger adulterated with wheat flour, corn flour, rice flour, and a mixture of the three flours.

Three classification algorithms were compared: Linear Discriminant Analysis (LDA), Support Vector Machines (SVMs), and Random Forest (RF). Models were developed using the training dataset (70% of the samples) and optimized by repeated 5-fold cross-validation to improve model robustness and minimize overfitting. Model performance was subsequently evaluated using the validation dataset (30% of the samples) based on the confusion matrix and the corresponding performance metrics, including accuracy, sensitivity, specificity, precision, and F1-score.

To investigate the influence of the spectral region and feature selection on classification performance, models were developed using the complete Vis–NIR spectrum (400–2500 nm), the visible region (400–700 nm), and the near-infrared region (700–2500 nm), both with and without Boruta feature selection. The classification performance obtained for the training and validation datasets is summarized in Table 1.

Among the evaluated classification approaches, Linear Discriminant Analysis (LDA) consistently provided the best classification performance. The highest accuracies were obtained for the D_283×4200_ (full Vis–NIR spectrum) and D_283×3600_ (NIR spectrum) datasets without Boruta feature selection, achieving validation accuracies above 95% and F1-scores exceeding 0.91. These results demonstrate that the spectral information contained in both the complete Vis–NIR spectrum and, particularly, the NIR region is sufficient to accurately discriminate authentic ginger samples from those adulterated with cereal flours. The comparable performance obtained using only the NIR region further indicates that most of the discriminatory information is associated with absorption bands related to the chemical composition of the samples rather than with colour differences captured in the visible region.

The excellent performance obtained using LDA is consistent with previous spectroscopic authentication studies on powdered foods [23], in which linear classification methods have often provided superior generalization compared with more complex machine learning algorithms when the spectral differences between authentic and adulterated samples are systematic [17]. Similar results were reported for paprika adulteration using portable NIR spectroscopy, where PLS-DA and related linear chemometric methods achieved highly reliable discrimination despite the complexity of the food matrix [15].

In contrast, both Support Vector Machine (SVM) and Random Forest (RF) models exhibited clear signs of overfitting [20]. Although both algorithms achieved very high accuracies in the training set, their performance decreased substantially when evaluated using the validation dataset, indicating limited generalization capability under the experimental conditions considered. This behaviour was particularly evident for the RF models, which reached perfect classification of the training data but considerably lower validation accuracies. Furthermore, models developed exclusively from the visible spectral region produced markedly poorer classification performance, especially for SVM and RF, confirming that colour information alone is insufficient to reliably distinguish adulterated ginger samples.

The application of Boruta feature selection did not improve the classification performance of any of the evaluated algorithms. On the contrary, a slight decrease in validation accuracy was observed in most cases, suggesting that the discarded wavelengths still contained useful complementary information for sample discrimination [24]. This finding indicates that, for the present dataset, the complete spectral information was more informative than the reduced feature subsets generated by Boruta.

Therefore, these results identify LDA as the most robust and reliable classification method for discriminating between authentic and adulterated ginger samples. Table 2 shows the confusion matrix obtained for the external validation set (samples with 8%, 18%, 28%, and 38% adulteration levels plus 4 pure ginger samples) using the LDA model developed from the D_283×3600_ (NIR) dataset without Boruta feature selection, illustrating the high classification accuracy achieved by the proposed approach.

### 2.2. Optimization of Partial Least Squares Regression (PLSR), Random Forest Regression (RFR), and Support Vector Regression (SVR) Models

The predictive performance of the regression models was evaluated using both the complete spectral datasets (D_283×4200_, D_283×601_, and D_283×3600_) and the corresponding reduced datasets generated by the Boruta feature-selection algorithm. Boruta is a wrapper method that identifies statistically relevant variables by comparing the importance of the original spectral variables with that of randomly generated shadow variables, retaining only those variables that contribute significantly to model performance [25].

PLSR, RFR, and SVR models were developed using the training dataset (70% of the samples) and optimized by repeated 5-fold cross-validation. Their predictive performance was subsequently evaluated using the validation dataset (30% of the samples). The regression results obtained for the different spectral regions and feature-selection strategies are summarized in Table 3.

Among all evaluated models, PLSR consistently provided the highest predictive performance, suggesting a linear relationship between the adulteration level and the spectroscopic data. The best results were obtained using the complete Vis–NIR spectrum (D_283×4200_) and the NIR spectrum (D_283×3600_) without Boruta feature selection, reaching validation coefficients of determination above 0.99, RMSE values below 1%, and RPD values exceeding 13. These results demonstrate the excellent capability of Vis–NIR spectroscopy combined with PLSR for the accurate quantification of flour adulteration in powdered ginger. Furthermore, the nearly identical performance obtained using the complete spectrum and only the NIR region indicates that the most relevant analytical information is contained within the near-infrared region, whereas the visible region contributes comparatively little to the predictive performance.

The effect of Boruta feature selection strongly depended on the regression algorithm. For PLSR, the elimination of variables consistently reduced predictive performance, with lower validation R^2^ values and higher prediction errors for both the complete spectrum and the NIR dataset. This behaviour suggests that PLSR efficiently exploits the covariance structure of the complete spectral dataset and therefore benefits from retaining the full spectral information rather than using a reduced subset of wavelengths.

Regarding the spectral variables retained by Boruta, the selected wavelengths for the regression models were distributed mainly in the visible region around 400–440 nm and across several near-infrared regions, broadly located near 900–1000 nm, 1050–1300 nm, 1500–1600 nm, 1700–1900 nm, and 2000–2350 nm, whereas for the classification models, the selected wavelengths were mainly concentrated near 1000–1150 nm and across several regions between 1650 and 2500 nm. The selected NIR regions are consistent with absorption domains associated with overtones and combination bands involving O–H, C–H, N–H, and C–O vibrations. In particular, the regions around 900–1000 nm are influenced by O–H and C–H absorptions associated with carbohydrates; the region around 1200 nm also contains C–H overtone contributions from carbohydrates; the 1500–1600 nm region includes O–H and N–H absorptions associated with starch and proteins; and the 1700–1900 nm region contains prominent C–H overtones and O–H/C–O combination bands from carbohydrates. The 2000–2350 nm region comprises several O–H/C–O, C–H, and N–H combination bands associated with carbohydrates, proteins, and related organic constituents.

In contrast, SVR exhibited the greatest sensitivity to feature selection. Without Boruta, the model developed using the NIR dataset showed excellent calibration statistics (R^2^ = 0.996) but very poor validation performance (R^2^ = 0.410; RPD = 1.06), indicating severe overfitting. This behaviour may be attributed to the curse of dimensionality associated with the large number of highly collinear variables, which can be partially mitigated by Boruta feature selection. Indeed, after applying Boruta feature selection, the predictive performance improved dramatically, reaching a validation R^2^ of 0.964 and an RPD of 4.81. A similar, although less pronounced, improvement was also observed for the complete Vis–NIR dataset. This behaviour suggests that SVR is considerably more sensitive to the presence of irrelevant and highly collinear spectral variables than PLSR or RFR in the current datasets. The removal of redundant wavelengths substantially improved the robustness of the kernel-based model, highlighting the importance of feature selection when nonlinear machine learning algorithms are applied to high-dimensional spectroscopic datasets.

Random Forest Regression showed the most stable behaviour among the evaluated algorithms. Validation R^2^ values remained consistently close to 0.97 for most spectral datasets, regardless of whether Boruta was applied, indicating that this ensemble learning method is comparatively insensitive to redundant spectral variables. This suggests that the decision tree splitting procedure naturally ignores uninformative variables even without the Boruta selection procedure. Nevertheless, its predictive accuracy remained consistently lower than that achieved by PLSR, suggesting that the additional flexibility of nonlinear tree-based models did not provide a significant advantage for this application.

The poorest predictive performance was obtained for models developed exclusively from the visible spectral region after Boruta feature selection. Under these conditions, all regression algorithms exhibited a marked reduction in predictive accuracy, with RPD values close to 2, indicating that the visible wavelengths alone do not contain sufficient compositional information for reliable quantitative determination of adulteration. These findings further confirm that the near-infrared region contains the most informative spectral features for modelling the levels of adulterants in powdered ginger.

Therefore, these results demonstrate that PLSR constitutes the most reliable and accurate regression approach for this application. Despite the greater flexibility of nonlinear machine learning algorithms, neither SVR nor RFR outperformed PLSR under the evaluated conditions. From a practical perspective, the combination of NIR spectroscopy and PLSR offers the most suitable compromise between prediction accuracy, robustness, computational simplicity, and ease of implementation, making it particularly attractive for routine quality control and food fraud detection in powdered ginger.

**Figure 1:**
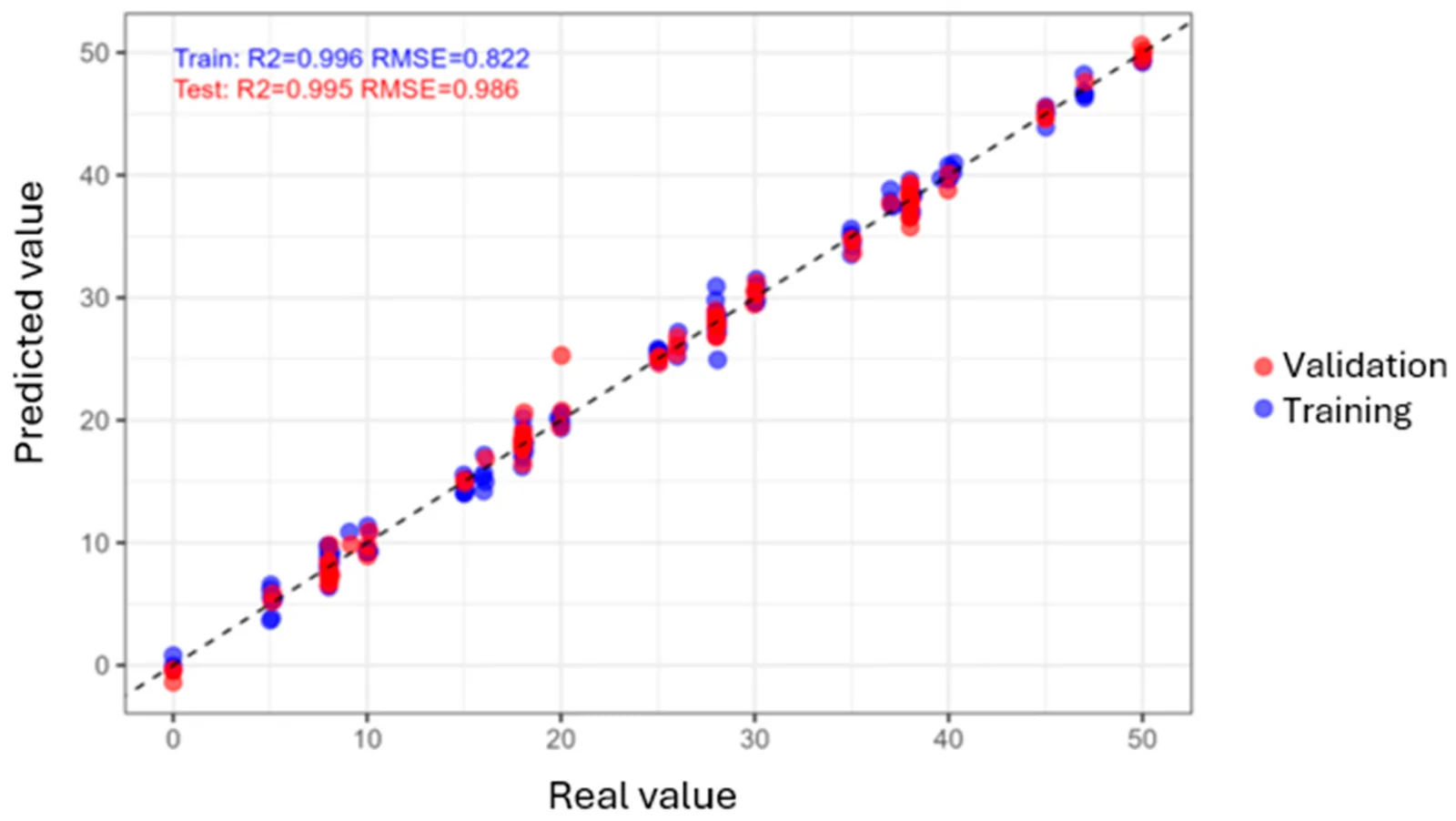
Relationship between predicted and reference ginger adulteration levels (%) obtained using the PLSR model developed from the NIR spectral dataset (D_283×3600_). Training and validation samples are represented separately, and the dashed 1:1 line represents ideal agreement between predicted and reference values. Deviations above and below this line correspond to overestimation and underestimation of the adulteration level, respectively.

Figure 1 illustrates the relationship between the reference and predicted adulteration levels obtained using the PLSR model developed from the D_283×3600_ (NIR region) dataset for both the training and validation sets. As expected from the statistical parameters reported in Table 3, an excellent agreement was observed between the experimental and predicted values. Most samples were closely distributed around the 1:1 reference line, yielding coefficients of determination of 0.996 and 0.995 for the training and validation datasets, respectively, with RMSE values below 1% in both cases.

The similar distribution of the training and validation samples indicates that the model generalizes well and shows no evidence of overfitting. Furthermore, no systematic overestimation or underestimation was observed throughout the investigated adulteration range (0–50%), and the prediction error remained remarkably uniform across the entire concentration interval. The absence of increasing residual dispersion at higher adulteration levels suggests that the model exhibits stable predictive performance over the full analytical range.

The close agreement between training and validation statistics confirms the robustness of the proposed PLSR model and demonstrates its suitability for the quantitative determination of cereal-flour adulteration in powdered ginger.

Previous spectroscopic studies have demonstrated the feasibility of rapid ginger adulteration detection and quantification, although differences in adulterants, analytical platforms, concentration ranges, chemometric approaches, and validation strategies should be considered when directly comparing their predictive performance (Table 4). Terouzi and Oussama applied ATR-FTMIR spectroscopy to quantify bean powder added to ginger over a 0–30% adulteration range, demonstrating the potential of vibrational spectroscopy for the quantitative assessment of ginger adulteration, although only a single adulterant was considered. More recently, Yu et al. [21] applied FT-NIR spectroscopy combined with chemometric and machine learning approaches to ginger adulterated with soybean, corn, and wheat flours, obtaining excellent classification and quantitative prediction performance.

The present study confirms this potential while extending the experimental and chemometric evaluation in several aspects. Four adulteration scenarios were considered, including wheat, corn, and rice flours individually and a mixture of the three cereal flours, and the visible, NIR, and combined Vis–NIR regions were systematically evaluated. Furthermore, linear and nonlinear approaches were directly compared for both classification and quantitative prediction, together with the effect of Boruta feature selection. Importantly, an additional external validation was performed using independently prepared samples containing adulteration levels (8, 18, 28, and 38%) that were not included among the concentration levels used for model development, thereby providing an additional assessment of model performance at previously unseen intermediate adulteration levels.

It must be noted that the ginger matrix used in this study was prepared by combining three different commercially available ginger powders; however, the natural variability of ginger should be considered when assessing the applicability of the developed models to market samples. Ginger powders from different cultivars, geographical origins, production batches, and processing conditions may differ in moisture, carbohydrate, phenolic, and volatile composition, as well as in particle-size distribution, all of which can influence Vis–NIR spectral responses. Further validation should consequently include a larger number of independent commercial ginger powders from different origins and manufacturers, with adulteration performed separately for each matrix. This would allow the influence of natural raw-material variability on model performance to be assessed.

## 3. Materials and Methods

### 3.1. Ginger Powder and Adulterants

Three commercially available ginger powders were used in this study: certified organic ginger powder Nature Vegan (Alpi, Roses, Spain), certified organic ginger powderNature (Alpi), and organic ginger powder Eco Powder (Frisafran, Corella, Spain). All products were purchased from local retailers in Spain.

Three commercial cereal flours, namely wheat, corn, and rice flour, were selected as adulterants because they are inexpensive ingredients that can be used to fraudulently dilute powdered ginger. All flours were purchased from local supermarkets in Spain. Corn flour was supplied by Harimsa (Murcia, Spain), while the remaining flours were obtained from commercial brands available in the Spanish market.

### 3.2. Preparation of Pure and Adulterated Samples

The ginger powders were purchased already milled. To incorporate variability among commercial products into the experimental matrix, 200 g of each of the three commercial ginger powders were thoroughly mixed, resulting in a homogeneous 600 g batch used as the authentic ginger matrix. This composite batch, rather than the three commercial ginger powders separately, was subsequently used for the preparation of all adulterated samples. Likewise, equal amounts (50 g) of wheat, corn, and rice flour were combined in a 1:1:1 (*w*/*w*) ratio to prepare a homogeneous 150 g flour mixture that was subsequently used as one of the adulterants.

Adulterated samples were prepared in duplicate. Consequently, four adulterated sample categories were generated: ginger adulterated with wheat flour (WF: 10 samples in duplicate), corn flour (CF: 10 samples in duplicate), rice flour (RF: 10 samples in duplicate), and the mixture of the three flours (WF-CF-RF: 10 samples in duplicate). Each of the three commercially available authentic ginger powders was measured in duplicate. Samples were gravimetrically prepared by mixing authentic ginger powder with each individual flour (wheat, corn, or rice) or with the flour mixture at adulteration levels of 5, 10, 15, 20, 25, 30, 35, 40, 45, and 50% (*w*/*w*). In addition, separate mixtures containing 8, 18, 28, and 38% (*w*/*w*) adulterant were independently prepared and used exclusively for external validation. These intermediate concentrations, which were not represented in the model development dataset, were intentionally selected to evaluate the ability of the models to predict unknown adulteration levels within the calibrated concentration range. Samples were identified according to the adulterant type, adulteration percentage, and replicate number. For example, a ginger sample adulterated with 5% corn flour was coded as CF5-1 or CF5-2. Therefore, 86 spectra were used for training and 32 spectra for validation.

### 3.3. Vis–NIR Spectral Acquisition

Diffuse reflectance spectra were acquired using a FOSS XDS Rapid Content^TM^ Analyzer (FOSS Analytical A/S, Hillerød, Denmark) equipped with a Vis–NIR monochromator. Spectra were recorded over the 400–2500 nm wavelength range with a data sampling interval of 0.5 nm, resulting in 4200 spectral variables per measurement. Each spectral measurement corresponded to the average of 32 scans automatically acquired by the instrument. Instrument background correction was performed using the standard internal reference procedure of the XDS system before sample measurement. Each sample was measured twice after repositioning, and the average of the two resulting spectra was used for subsequent chemometric analysis to minimize instrumental and sampling variability.

### 3.4. Spectral Preprocessing

Prior to chemometric analysis, spectral data were preprocessed to reduce instrumental noise, minimize baseline variations, and enhance spectral features associated with compositional differences among samples. Savitzky–Golay smoothing followed by first-derivative transformation was applied using a nine-point window and a second-order polynomial. These preprocessing parameters were kept constant throughout the study and were not subjected to an optimization procedure. This preprocessing strategy was selected to improve spectral resolution while reducing scattering effects and enhancing subtle absorption features associated with adulteration. The preprocessed spectra were subsequently used for the development of the classification and regression models.

### 3.5. Chemometric Analysis

Chemometric analyses were performed to classify samples according to the adulterant type and to quantify the adulteration level. Three supervised classification algorithms were evaluated: Linear Discriminant Analysis (LDA), Support Vector Machine (SVM), and Random Forest (RF). Quantitative prediction models were developed using Partial Least Squares Regression (PLSR), Support Vector Regression (SVR), and Random Forest Regression (RFR).

The model-development dataset was randomly divided once into training (70%) and validation (30%) subsets. Model optimization was performed within the training set using repeated 5-fold cross-validation. Final model performance was first assessed using the 30% validation set. In addition, an independent external validation was performed using separately prepared samples containing 8, 18, 28, and 38% (*w*/*w*) adulterant. These concentration levels were not included in the model-development dataset and therefore provided an additional assessment of the model’s predictive capability using independently prepared samples at previously unseen adulteration levels.

For regression analysis, variable selection was performed using the Boruta algorithm, with a maximum of 500 iterations and a significance threshold of *p* < 0.01. Hyperparameter optimization was carried out through grid search combined with 5-fold cross-validation, using RMSE as the selection criterion. For the PLS model, the number of latent variables was optimized by evaluating 15 candidate values. For the SVM model, using a radial kernel, the cost (C) and gamma parameters were tuned in two successive grid search stages, initially exploring values between 2^−5^ and 2^5^ and then refining the search within a ±50% range around the best values obtained. For Random Forest, the number of candidate variables per split (mtry) was optimized through grid search, while the number of trees (ntree) was kept fixed at 500.

In the case of the classification study, variable selection using Boruta was performed with the same configuration as in the regression models, using a maximum of 500 iterations and a significance threshold of *p* < 0.01. Hyperparameters were optimized through grid search combined with 5-fold cross-validation, using accuracy as the selection criterion. For the SVM model, using a radial kernel, the cost (C) and gamma parameters were tuned following the same two-stage grid search scheme described for the regression model. For Random Forest, mtry was optimized through grid search, while ntree was kept fixed at 500. The LDA model, having no tunable hyperparameters, was trained directly under the same cross-validation scheme used for the other models.

The predictive performance of the regression models was evaluated using the coefficient of determination (R^2^), root mean square error (RMSE), and ratio of performance to deviation (RPD). The coefficient of determination describes the proportion of variance explained by the model, whereas RMSE estimates the average prediction error expressed in the same units as the reference values. RPD was calculated as the ratio between the standard deviations of the reference measurements. For validation, the standard deviation of the reference adulteration levels was 13.42%.

All spectral preprocessing, chemometric analyses, and graphical representations were performed using R software (version 4.5.1; R Foundation for Statistical Computing, Vienna, Austria) employing the packages prospectr, caret, pls, e1071, randomForest, vip, and ggplot2.

The optimized model parameters used in the chemometric analysis are summarized in Table 5 and Table 6 for classification and regression, respectively.

## 4. Conclusions

This study demonstrated that Vis–NIR spectroscopy provides sufficient spectral information for the reliable detection and quantification of powdered ginger adulteration with different cereal flours. The developed chemometric models successfully discriminated between authentic and adulterated samples while accurately estimating the adulteration level over a wide concentration range. Among the evaluated regression approaches, Partial Least Squares Regression (PLSR) combined with the NIR spectral region provided the highest predictive performance, achieving coefficients of determination above 0.99 and prediction errors below 1%, thereby confirming its suitability for quantitative authenticity assessment.

The proposed methodology represents a rapid, non-destructive, reagent-free, and cost-effective alternative to conventional analytical techniques for routine quality control of powdered ginger. The results also demonstrate that increasing model complexity does not necessarily improve predictive performance, as the conventional PLSR approach outperformed or matched more sophisticated machine learning algorithms under the conditions evaluated.

## Figures and Tables

**Table 1:** Training and validation performance of SVM, Random Forest (RF), and Linear Discriminant Analysis (LDA) classification models developed using different spectral ranges (Vis–NIR, Vis, and NIR) with and without Boruta feature selection.

Spectral Dataset	Model	Training Set	Validation Set				
		Accuracy	Accuracy	Sensitivity	Specificity	Precision	F1
**D_283×4200_** **(Vis-NIR)**	**SVM_Radial**	0.8392	0.4286	0.4221	0.8806	0.5009	0.4377
	**RF**	1.0000	0.3452	0.3664	0.8644	0.4448	0.3880
	**LDA**	1.0000	0.9524	0.8870	0.9899	0.9644	0.9114
**D_283×4200_** **(Vis-NIR + Boruta)**	**SVM_Radial**	0.3719	0.3214	0.4093	0.8580	0.3544	0.3894
	**RF**	1.0000	0.2976	0.3981	0.8547	0.3150	0.4100
	**LDA**	0.6231	0.5238	0.5974	0.9020	0.5047	0.5268
**D_283×601_** **(Vis)**	**SVM_Radial**	0.0905	0.2976	0.3796	0.8510	0.5183	0.5910
	**RF**	1.0000	0.1905	0.2312	0.8315	0.2275	0.2278
	**LDA**	1.0000	0.7024	0.6024	0.9380	0.7157	0.7128
**D_283×601_** **(Vis + Boruta)**	**SVM_Radial**	0.0905	0.2976	0.3796	0.8510	0.5183	0.5910
	**RF**	1.0000	0.1905	0.2312	0.8315	0.2275	0.2278
	**LDA**	1.0000	0.7024	0.6024	0.9380	0.7157	0.7128
**D_283×3600_** **(NIR)**	**SVM_Radial**	0.7889	0.5119	0.4272	0.8968	0.7087	0.5940
	**RF**	1.0000	0.8929	0.8390	0.9780	0.9130	0.8585
	**LDA**	1.0000	0.9643	0.9722	0.9924	0.9762	0.9720
**D_283×3600_** **(NIR + Boruta)**	**SVM_Radial**	0.5930	0.5119	0.5321	0.8997	0.5293	0.4429
	**RF**	1.0000	0.8810	0.8262	0.9754	0.9020	0.8471
	**LDA**	1.0000	0.8452	0.8707	0.9677	0.8717	0.8708

**Table 2:** Confusion matrix of the independent external validation set obtained using the LDA model developed from the NIR spectral dataset without Boruta feature selection.

	Reference					
		WF-CF-RF	RF	CF	WF	G
Prediction	**WF-CF-RF**	16	0	3	0	0
	**RF**	0	16	0	0	0
	**CF**	0	0	13	0	0
	**WF**	0	0	0	16	0
	**G**	0	0	0	0	4

**Table 3:** Training and validation performance of PLS, SVM, and RF regression models for ginger adulteration quantification using different spectral datasets with and without Boruta feature selection.

Data Set	Model	Training		Validation			
		R^2^	RMSE	R^2^	RMSE	Bias	RPD
**D_283×4200_ (Total Spectrum)**	**PLS**	0.9960	0.8355	0.9952	0.9268	−0.07	14.48
	**SVM**	0.9967	1.2532	0.8530	6.0395	−0.48	2.22
	**RF**	0.994	1.0709	0.9647	2.6316	−0.19	5.10
**D_283×4200_ (Total Spectrum) + Boruta**	**PLS**	0.9852	1.5982	0.9827	1.7560	−0.04	7.64
	**SVM**	0.9934	1.0802	0.9846	1.6872	0.22	7.95
	**RF**	0.9939	1.0641	0.9686	2.4747	0.09	5.42
**D_283×601_ (Visible Spectrum)**	**PLS**	0.987	1.4996	0.9852	1.6382	0.20	8.19
	**SVM**	0.9918	1.2064	0.9834	1.7435	0.16	7.70
	**RF**	0.9915	1.2387	0.968	2.4945	−0.08	5.38
**D_283×601_ (Visible Spectrum) + Boruta**	**PLS**	0.8091	5.7436	0.8062	5.8936	0.50	2.28
	**SVM**	0.8874	4.4707	0.8670	4.9891	0.67	2.69
	**RF**	0.9680	2.3894	0.8396	5.3629	−0.11	2.50
**D_283×3600_ (NIR Spectrum)**	**PLS**	0.9961	0.8220	0.9946	0.9860	0.02	13.61
	**SVM**	0.9961	1.6388	0.4097	12.6665	0.23	1.06
	**RF**	0.9939	1.1216	0.9652	2.7003	−0.35	4.97
**D_283×3600_ (NIR Spectrum) + Boruta**	**PLS**	0.9869	1.5038	0.9764	2.1132	−0.4	6.35
	**SVM**	0.9947	1.0472	0.9637	2.7930	0.2	4.81
	**RF**	0.9947	1.0496	0.9717	2.4956	−0.33	5.38

**Table 4:** Comparison of spectroscopic approaches previously reported for ginger adulteration detection and quantification with the present study.

Study	Adulterant(s)	Range	Spectroscopic Technique	Models	Reported Performance
Terouzi & Oussama, 2016 [26]	Bean powder	0–30%	ATR-FTMIR	PLSR	R^2^ ≈ 0.99; detection limit ≈ 3.3%
Yu et al., 2022 [21]	Soybean, corn and wheat flour	10–50%	FT-NIR	PCA, PLS-DA/ML, PLSR	Classification up to 100%; RPD up to 14.61
Present study	Wheat, corn, rice and three-flour mixture	5–50%; external validation at 8, 18, 28, and 38%	Vis–NIR 400–2500 nm	LDA, SVM, RF; PLSR, SVR, RFR	LDA accuracy 96.4%; PLSR validation R^2^ = 0.9946; RMSE = 0.986%; RPD = 13.61

**Table 5:** Optimized hyperparameters of the classification models and number of spectral variables selected by Boruta for the different Vis–NIR spectral datasets.

Spectral Dataset	Model	Models Optimization	
		Optimized Hyperparameters	Number of Variables Selected by Boruta
**D_283×4200_** **(Vis-NIR)**	**SVM_Radial**	C = 40; γ = 0.015625	-
	**RF**	Ntree = 500; Mtry = 91	-
	**LDA**	Not applicable (no tunable hyperparameters)	-
**D_283×4200_** **(Vis-NIR + Boruta)**	**SVM_Radial**	C = 0.015625; γ = 1.5	15
	**RF**	Ntree = 500; Mtry = 15	15
	**LDA**	Not applicable (no tunable hyperparameters)	15
**D_283×601_** **(Vis)**	**SVM_Radial**	C = 16; γ = 0.03125	-
	**RF**	Ntree = 500; Mtry = 2	-
	**LDA**	Not applicable (no tunable hyperparameters)	-
**D_283×601_** **(Vis + Boruta)**	**SVM_Radial**	C = 16; γ = 0.03125	10
	**RF**	Ntree = 500; Mtry = 2	10
	**LDA**	Not applicable (no tunable hyperparameters)	10
**D_283×3600_** **(NIR)**	**SVM_Radial**	C = 4; γ = 0.015625	-
	**RF**	Ntree = 500; Mtry = 551	-
	**LDA**	Not applicable (no tunable hyperparameters)	-
**D_283×3600_** **(NIR + Boruta)**	**SVM_Radial**	C = 0.15625; γ = 0.0234375	223
	**RF**	Ntree = 500; Mtry = 2	223
	**LDA**	Not applicable (no tunable hyperparameters)	223

**Table 6:** Optimized hyperparameters of the regression models and number of spectral variables selected by Boruta for the different Vis–NIR spectral datasets.

Spectral Dataset	Model	Models Optimization	
		Optimized Hyperparameters	Number of Variables Selected by Boruta
**D_283×4200_ (Total Spectrum)**	**PLS**	Number of latent variables (LVs) = 15	-
	**SVR**	C = 2; γ = 0.015625	-
	**RFR**	Ntree = 500; Mtry = 25	-
**D_283×4200_ (Total Spectrum) + Boruta**	**PLS**	Number of latent variables (LVs) = 15	205
	**SVR**	C = 48; γ = 0.015625	205
	**RFR**	Ntree = 500; Mtry = 2	205
**D_283×601_ (Visible Spectrum)**	**PLS**	Number of latent variables (LVs) = 10	-
	**SVR**	C = 12; γ = 0.015625	-
	**RFR**	Ntree = 500; Mtry = 7	-
**D_283×601_ (Visible Spectrum) + Boruta**	**PLS**	Number of latent variables (LVs) = 4	61
	**SVR**	C = 6; γ = 0.125	61
	**RFR**	Ntree = 500; Mtry = 41	61
**D_283×3600_ (NIR Spectrum)**	**PLS**	Number of latent variables (LVs) = 13	-
	**SVR**	C = 1.5; γ = 0.015625	-
	**RFR**	Ntree = 500; Mtry = 128	-
**D_283×3600_ (NIR Spectrum) + Boruta**	**PLS**	Number of latent variables (LVs) = 11	254
	**SVR**	C = 2; γ = 0.015625	254
	**RFR**	Ntree = 500; Mtry = 2	254

## Data Availability

Authors will share the data used in this manuscript upon request.
